# Knocking Out the Transcription Factor OsNAC092 Promoted Rice Drought Tolerance

**DOI:** 10.3390/biology11121830

**Published:** 2022-12-15

**Authors:** Bo Wang, Yiheng Wang, Wancong Yu, Luping Wang, Qingkuo Lan, Yong Wang, Chengbin Chen, Yong Zhang

**Affiliations:** 1 Institute of Germplasm Resources and Biotechnology, Tianjin Academy of Agricultural Sciences, Tianjin 300071, China; 2 College of Life Sciences, Nankai University, Tianjin 300071, China; 3 Department of Biotechnology, School of Life Sciences and Technology, Center for Informational Biology, University of Electronic Science and Technology of China, Chengdu 610054, China

**Keywords:** rice, NAC transcription factor, CRISPR-Cas9, drought stress, transcriptome analysis

## Abstract

**Simple Summary:**

Rice is one of the most globally important food crops. More than half of the world’s population depends on rice as a source of food. In recent years, with the deterioration of the ecological environment and the frequent occurrence of extreme weather, drought, high temperature, cold damage, salinity, and other stress factors have become increasingly prominent, seriously affecting rice production. Drought is one of the factors that seriously affects the growth and yield of rice. After cross breeding, molecular breeding slowly stepped onto the historical stage. Our experiment aimed to study the gene function of members of the transcription family, thus providing ideas and theoretical support for molecular breeding under stress.

**Abstract:**

Environmental drought stress threatens rice production. Previous studies have reported that related NAC (NAM, ATAF1/2, and CUC) transcription factors play an important role in drought stress. Herein, we identified and characterized OsNAC09*2*, encoding an NAC transcription factor that is highly expressed and induced during drought tolerance. *OsNAC092* knockout lines created using the clustered regularly interspaced palindromic repeats (CRISPR)-associated protein 9 (Cas9) system exhibited increased drought resistance in rice. RNA sequencing showed that the knockout of *OsNAC092* caused a global expression change, and differential gene expression is chiefly associated with “response to light stimulus,” “MAPK signaling pathway,” “plant hormone signal transduction,” “response to oxidative stress,” “photosynthesis,” and “water deprivation.” In addition, the antioxidants and enzyme activities of the redox response were significantly increased. *OsNAC092* mutant rice exhibited a higher ability to scavenge more ROS and maintained a high GSH/GSSG ratio and redox level under drought stress, which could protect cells from oxidant stress, revealing the importance of OsNAC092 in the rice’s response to abiotic stress. Functional analysis of OsNAC092 will be useful to explore many rice resistance genes in molecular breeding to aid in the development of modern agriculture.

## 1. Introduction

Over half of the world’s population relies on rice (*Oryza sativa* L.) as its staple food crop [1,2]. During plant growth and development, various abiotic stresses may be encountered, of which drought is one of the main reasons for rice yield reduction [3,4]. Rice mounts physiological, biochemical, cellular, and molecular responses to external environmental stresses to aid in its survival [5,6].

Transcription factors are a class of regulatory proteins that regulate gene expression [7,8]. In plants, one of the largest transcription factor (TF) families is the NAC (NAM, ATAF1/2, CUC2) family [9,10]. During the growth and development of plants, NAC TFs regulate a variety of biological processes. For example, knockout of wheat *TaNAC071*-*A* decreased wheat’s tolerance to drought, whereas *TaNAC071*-*A* overexpression increased water use efficiency to markedly enhance drought tolerance. This enhanced water saving mechanism mitigated yield losses caused by water scarcity [11]. In addition, soybean NAC-type transcription factor gene *GmNAC20* was transformed into the rice genome, which improved both cold and salinity tolerance [12]. Moreover, the rice *osnac016* mutant showed increased tolerance to drought stress, which was associated with increased stomatal closure and a reduced water loss rate under exogenous abscisic acid (ABA) [13]. Furthermore, rice with a knockout of *OsNAC041* became salt sensitive, which was associated with altered expression of reactive oxygen species (ROS)-related genes [14]. Finally, clustered regularly interspaced palindromic repeats (CRISPR)-associated protein 9 (Cas9)-mediated knockout of *OsNAC006* increased rice sensitivity to drought and heat via alterations to many stress-response pathways [15].

Under unfavorable growth environments, ROS accumulate to expose plants to oxidative damage [16]. The changes in redox status caused by the accumulation of ROS molecules in the mitochondria, chloroplasts, and peroxisomes can be transmitted to the nucleus through the organelle–nucleus retrograde signaling pathway, resulting in differences in the expression of many genes. In this process, glutathione can scavenge the accumulation of ROS through direct or indirect action, maintaining the homeostasis of the intracellular environment [17]. Glutathione participates in the glutathione-ascorbic acid cycle, reducing hydrogen peroxide by oxidizing itself. The conversion between reduced glutathione (GSH) and oxidized glutathione (GSSG) is carried out by three catalytic enzymes, including glutathione peroxidase (GPX), glutathione reductase (GR), and glutathione S-transferase (GST). Moreover, in addition to participating in the glutathione-ascorbic acid cycle, glutathione can also reduce other types of peroxides, such as membranous peroxides, glyoxal, and herbicide resistance through GPX [18]. In addition, GSH can not only directly scavenge ROS accumulated in plants under adverse environments, but also activate multiple downstream defense systems to indirectly protect cells from damage [19,20]. In an extension to the study, in order to decipher the role of *OsNAC092* (Os06g0675600) in drought, we first comprehensively analyzed the *OsNAC092* expression profiles in various stress conditions. Secondly, we constructed the mutants structure using the CRISPR-Cas9 vector and transformed it into rice. Through drought stress, we demonstrated that the mutant plants exhibited enhanced tolerance and performed better under drought stress than WT at the phenotypic, physiological, and molecular levels. From our analyses, we report that *OsNAC092* as a transcription factor plays a role in drought stress by regulating the related genes involved in drought tolerance. Our findings have significant implications for the future molecular breeding of rice.

## 2. Materials and Methods

### 2.1. Plants and their Growth Conditions

Seeds of wild-type rice (Nipponbare, *Oryza sativa* L. japonica) and *osnac092* mutants were cultured on hormone-free 1/2 MS agar medium containing 30 g L^−1^ sucrose and 6 g L^−1^ agar after surface sterilization with 1% NaClO. Cultures were maintained at 28 °C under a 16 h light/8 h dark photoperiod in a light incubator fitted with 3000 lux lamps. Mutant plants were transformed using the Agrobacterium-mediated seed callus method. After the infection of the callus with Agrobacterium at OD600 = 0.1, the callus was cultured in the dark at 25 °C for 3 d. The callus was then washed six times with sterile water, and the excess water was wiped off with filter paper to dry. The culture conditions included a continuous light culture at 32 °C for 2 weeks. Regeneration culturing was carried out for 4 weeks, and the medium was changed every two weeks. The differentiated regenerated seedlings were transferred to a rooting medium, with culture conditions of 28 °C in a light incubator, 16 h light/8 h dark. When the obtained regenerated plants grew to about 12–13 cm, the positive transformed plants were then grown in soil for further observation. After propagation to the T2 generation and after identification, the stress experiment was carried out.

### 2.2. Drought Stress Treatment

For drought stress phenotypic observations during germination, after tissue culture disinfection, the seeds were planted directly on 1/2 strength MS medium with mannitol at 150 mM. The germination situation was observed after 7 days of treatment. The seeds on the medium were placed in a 28 °C lighted incubator with a 16 h/8 h photoperiod.

To analyze the drought tolerance of *osnac092* mutant plants at the seedling stage, plants were selected growing on a high osmolality media (mannitol 150 mM) to carry out drought stress. During the eighth week, plants were stressed by water withdrawal, at which time other physiological indicators were measured in leaves from the same location of at least three randomly selected plants per treatment. Eight-week-old WT and mutants with consistent growth were selected for 14 days of drought stress. Drought stress treatments were performed under a 16 h/8 h photoperiod in a growth chamber at 28 °C.

Both well-watered and drought-stressed plant pots were saturated with water and left overnight to drain before drought stress began. Finally, plants at a consistent developmental stage were selected for stress treatment.

### 2.3. Promoter Prediction Analysis

In this experiment, the RAP-DB database was used to query the *OsNAC092* promoter sequence. The *OsNAC092* promoter sequence occurred 2000 bp upstream of the initiation codon (ATG). The online website (https://www.dna.affrc.go.jp/PLACE) (accessed on 1 January 2019) was used to predict the possible binding sites in the *OsNAC092* promoter (Supplementary Materials Table S1).

### 2.4. OsNAC092 Expression Profile Analysis

To study the organ expression of *OsNAC092*, WT seeds were germinated in soil for expression detection. Four-week-old WT seedlings were exposed to a low temperature (4 °C), heat (42 °C), PEG 6000 (20%, w/v), NaCl (200 mM), H_2_O_2_ (1%), IAA (indole-3-acetic acid) (100 mM), ABA (abscisic acid) (100 mM), and GA_3_ (gibberellic acid) (100 mM). At different time periods after treatment, the plants were washed with sterile water and dried. The whole plant tissues were then subjected to RNA extraction, followed by qRT-PCR analysis.

The roots, leaves, and sheaths were sampled from 4-week-old seedlings, and roots, stems, and leaves were sampled from 8-week-old rice seedlings. Total RNA was isolated from these samples using the modified Trizol Reagent (Invitrogen, Waltham, MA, USA) following the supplier’s protocol. The RNA samples were the subjected to qRT-PCR to assess *OsNAC092* expression in the WT plants, employing the primers shown in Supplementary Materials Table S2. The actin gene was used as the internal control. Three biological replicates (i.e., three independent plants in each treatment group) were assessed to guarantee reproducibility.

### 2.5. Targeted Mutagenesis of OsNAC092

*OsNAC092* mutants were generated using the CRISPR-Cas9 method with the pZHY988 backbone vector. The online tool CRISPR-P (http://crispr.hzau.edu.cn/CRISPR2) (accessed on 14 December 2022) was used to design the sgRNA. Potential off-target sites of the sgRNA were also predicted using CRISP-P. Primers were then designed for the sequence analysis of off-target sites (Supplementary Materials Table S3). We screened the plants that were positive for the transgene using DNA sequencing to determine whether they were mutants and to determine the specific mutation type [21,22].

### 2.6. Physiological Measurements

Seeds of the WT and mutants were grown in pots to the 8-week-old seedling stage, followed by the induction of 14 days of drought stress. To measure SOD, POD, CAT, and MDA activities, 0.5 g of fresh leaves were ground into a powder with 5 mL of PBS (pH 7.8). After centrifugation, the supernatant (enzyme solution) was removed to test tubes and stored at 4 °C during a short period (less than 15 min) until processing. SOD, POD, CAT, and MDA activities were measured as described in previously published articles [14]. Each experiment was repeated three times with three separate plants. Reduced glutathione (article No: A006-2-1), total glutathione (article No: A061-1-2), ascorbic acid content (article No: A122-1-1), glutathione peroxidase activity (A005-1-2), glutathione reductase activity (article No: A062-1-1) and glutathione S-transferase (article No: A004-1-1) were all assessed according to the kit (Nanjing Jiancheng, China); hydrogen peroxide content was also evaluated according to the kit (Tiangen, China). The content of oxidized glutathione is calculated according to the formula: (total glutathione-reduced glutathione)/2.

### 2.7. Transcriptome Analysis

As the materials, RNA-seq analysis used WT, *osnac092* (T/T), and *osnac092* (−1/−1) mutant plants grown for 10 weeks under normal conditions, or 8 weeks under normal conditions, followed by 2 weeks under drought treatment. A mixed sample was formed by combining three T2 seedlings. The Beijing Genomics Institute (Shenzhen, China) carried out the RNA-seq sequencing and analyses of the samples. qRT-PCR was used to verify the accuracy of the RNA-seq data using 10 randomly selected genes with significantly different expression profiles. The qRT-PCR data were normalized against the expression of actin. The assays were performed under identical conditions, in triplicate.

### 2.8. Data Analysis

This project uses the BGISEQ-500 platform(Guang Dong, China) for detection and removes low-quality, linker contamination, and reads with high N content of unknown origin through data filtering to ensure the reliability of the results. After obtaining the clean reads, HISAT was used to align the clean reads to the reference genome sequence. The comparison rate of all samples reached more than 97%, and the uniform comparison rate indicated that the data were comparable between samples. Later, Bowtie2 (Guang Dong, China) was used to align the clean reads to the reference sequence, and then RSEM3\ (Guang Dong, China)was used to calculate the expression levels of the genes and the transcripts. Finally, the gene expression between samples and between groups, the gene expression cluster analysis, and the differentially expressed gene detection were completed.

The data obtained by sequencing are called raw reads or raw data, and the raw reads are subsequently subjected to quality control (QC) to determine whether the sequencing data is suitable for further analysis. After quality control, the filtered clean reads were aligned to the reference sequence. After the alignment, it is determined whether the alignment result passes the second quality control (QC of alignment) by counting the alignment rate and the distribution of reads on the reference sequence. The samples in this experiment were all tested, and the quantitative analysis of the genes and various analyses based on gene expression levels (principal component, correlation, differential gene screening, etc.) were carried out in the later stage, and the GO function was performed on the differentially expressed genes among the selected samples. More in-depth mining analyses were also employed, such as significant enrichment analysis, pathway significant enrichment analysis, clustering, protein interaction networks, transcription factors, SNP and InDel, and differentially spliced gene detection.

## 3. Results

### 3.1. Expression Profiling of OsNAC092

**Figure 1:**
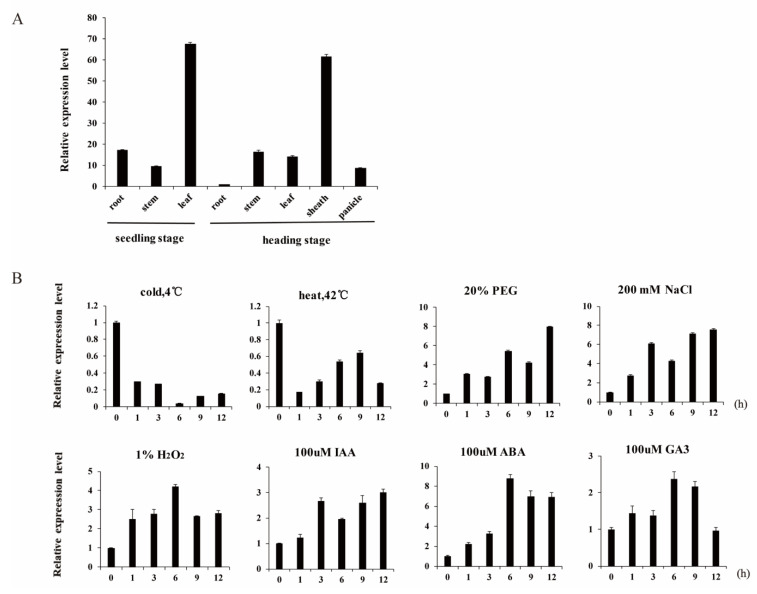
Expression pattern analysis of *OsNAC092*. (**A**). Tissue-specific expression pattern of *OsNAC092*. The X axis represents different sampling positions. Root, sheath and leaf samples at the seedling stage. Root, stem, sheath, leaf and panicle samples at the reproductive growth stage. (**B**). Expression levels of *OsNAC092* under various abiotic stresses and hormone treatments. Four-week-old seedlings were subjected to treatment with cold (4 °C), heat (42 °C), PEG 6000 (20%, w/v), NaCl (200 mm), H2O2 (1%), IAA (100 μm), ABA (100 μm) and GA3 (100 μm). X axis values represent time after starting treatments. The relative expression level of OsNAC092 was measured by RT-qPCR at the indicated times. Error bars indicate SE based on three independent biological replicates.

OsNAC092 expression in eight representative rice tissues (seedling leaves, stems, and roots, as well as heading stage panicles, leaves, sheaths, stems, and roots) was assessed using quantitative real-time reverse transcription PCR (qRT-PCR) (Figure 1A). Heading refers to a fully developed spike of cereal crops that stretches out the top leaves, with the elongation of the stems. At the age of 8 weeks, rice seedlings enter the heading stage. The results indicated that *OsNAC092* was mainly expressed in leaves in the seedling stage and in the sheath at the heading stage, at much higher levels than in the roots and stems.

The qRT-PCR results showed that *OsNAC092* could respond to adverse stress, with the most obvious response (increased expression) to drought and salt. Overall, temperature change inhibited the expression of *OsNAC092*; however, after an initial decrease, some recovery of expression was observed at 42 °C. Plant hormones (indole acetic acid—IAA), ABA, gibberellin A3 (GA3), and H_2_O_2_ treatments induced the expression of *OsNAC092* (Figure 1B).

We performed a binding site prediction analysis for the promoter region 2000 bp before the start codon for the WRKY DNA-binding protein 71 (WRKY71), MYB domain protein 2 (MYB2), early responsive to dehydration 1 (ERD1), Dof-type zinc finger DNA-binding family protein (DOF), and MYC domain protein, which are related to growth and stress (Supplementary Materials Table S1).

### 3.2. OsNAC092 Targeted Mutagenesis

**Figure 2:**
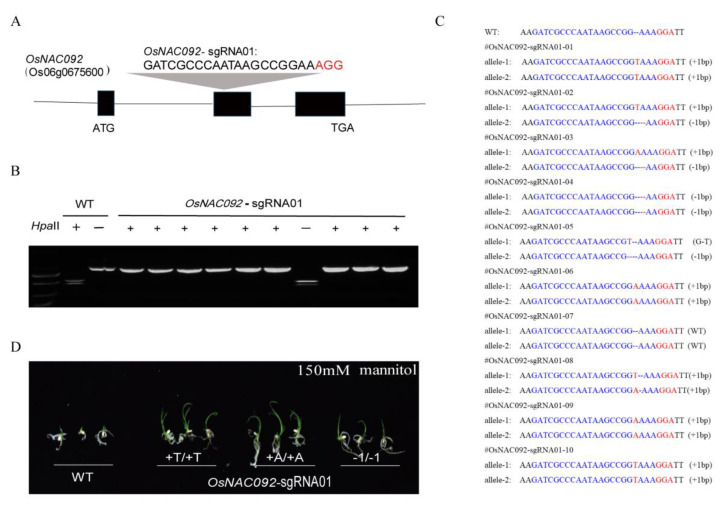
Using CRISPR-Cas9 system to create mutants. (**A**). Design of the sgRNA site for *OsNAC092* exons. (**B**). *osnac092* T0 genotype digestion test results. (**C**). Sanger sequencing results of *osnac092* T0 generation. (**D**). MS + 150 mmmol mannitol germination results under simulated drought treatment. *osnac092* (T/T) and *osnac092* (A/A), insertion of a single base pair (+1 bp/+1 bp). *osnac092* (−1/−1), a single base pair deletion (−1 bp/−1 bp).

*OsNAC092* loss of function lines were created using the CRISPR-Cas9 system. The single guide RNA (sgRNA) was designed close to the 5′ end of the second exon of *OsNAC092*, which was ligated into the CRISPR-Cas9 T-DNA vector, followed by transformation into rice calli (Figure 2A). Ten regenerated rice plants were tested in the experiment, yielding a transgene positive rate of 100% (10/10). The preliminary detection of mutants was carried out using restriction enzyme digestion, followed by Sanger sequencing to verify the mutated genotype. A total of 10 T0 generation transgenic positive plants were detected using the PCR-RFLP method. The results of enzyme digestion showed that 9 plants were mutated, and the mutation efficiency reached 90% (Figure 2B). The sequencing results showed that the mutation types in these mutants were all homozygous mutations. *OsNAC092*-sgRNA01-01, 06, 08, 09, and 10 were all biallelic insertions of 1 bp, and the inserted base was T or A. *OsNAC092*- sgRNA01-04 is a biallelic deletion of 1 bp, and *OsNAC092*-sgRNA01-05 has a G substitution of T in one strand and a single nucleotide deletion in the other strand. The analysis result showed that the mutations included the insertion of a single base pair (+1 bp/+1 bp), *osnac092* (T/T) and *osnac092* (A/A), and a single base pair deletion (−1 bp/−1 bp), osn*ac092* (−1/−1) (Figure 2C). The amino acid expression analysis of *osnac092* showed that the OsNAC092 of the mutants would change, and the transcription would be terminated in advance; thus, the core structure cannot be formed, and the gene would lose its function (Supplementary Materials Figure S1). Seed propagation was carried out to the T2 generation for subsequent experiments. At the same time, possible off-target sites were detected using Sanger sequencing of the PCR products (Supplementary Materials Table S2). Moreover, germinating seeds were treated with 150 mM mannitol to simulate drought stress. The results showed that the *osnac092* plants were more tolerant to drought stress at the germination stage than the WT (Figure 2D).

### 3.3. Loss of Function of OsNAC092 Increases Tolerance of Rice Seedlings to Drought Stress

**Figure 3:**
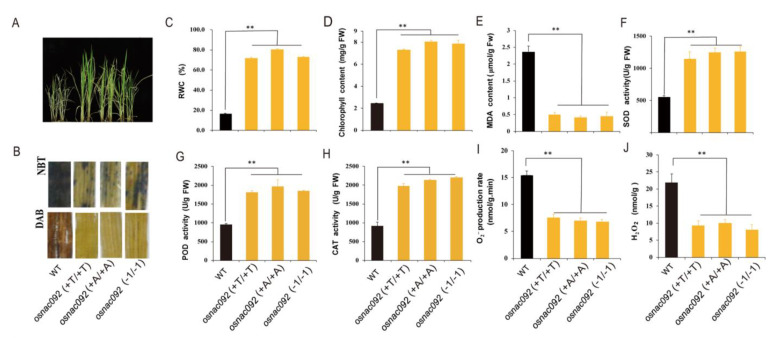
Phenotypic of *osnac092* mutants and determination of physiological indexes after drought stress. (**A**). Phenotypic of *osnac092* mutants after drought stress. Eight-week-old WT and mutants were selected for 14 days drought stress. (**B**). NBT and DAB staining result. (**C**). relative water content. (**D**). chlorophyll content. (**E**). MDA content. (**F**). SOD activity. (**G**). POD activity. (**H**). CAT activity. (**I**). O2–content production rate determination. (**J**). H2O2 content determination. The detection of relevant physiological indicators is also 8-week-old + 14 days drought treatment. ** represent significant differences *p* < 0.01 compared with wide type.

We observed that the *osnac092* mutants exhibited better drought resistance than the WT plants. Eight-week-old WT and *osnac092* plants with consistent growth were selected for drought stress testing. After 14 days of drought treatment, the WT plants began to wilt severely, while the *osnac092* mutant line was less affected (Figure 3A). Overall, the O_2_^−^ content in the WT was 2–3 times higher than that in the *osnac092* (Figure 3I); the accumulated H_2_O_2_ content was 2–2.5 times higher than that of the *osnac092* (Figure 3J), and the results of staining with Nitro Blue Tetrazolium (NBT) and 3,3’-diaminobenzidine (DAB) were consistent with the assay results (Figure 3B). After drought stress, the water content of the *osnac092* mutant was 3.5–5 times higher than that of the WT (Figure 3C). At the same time, the chlorophyll content of the *osnac092* mutant line was 3–4 times higher than that of the WT (Figure 3D). The content of MDA in *osnac092* was significantly lower than that of the WT, and the activities of superoxide dismutase (SOD), peroxidase (POD), and catalase (CAT) in *osnac092* were significantly higher than those of the WT, indicating that *osnac092* plants experienced less oxidative damage under drought stress (Figure 3E–H).

### 3.4. OsNAC092 Knockout Changed Rice Global Gene Expression

To study the mechanism of action by which the knockout of *OsNAC092* enhances the drought tolerance of rice, WT, *osnac092* (T/T), and *osnac092* (−1/−1) were grown for 10 weeks in normal nutrient soil and for 8 weeks under normal growth conditions +2 weeks of drought stress. The plant materials were then subjected to RNA-sequencing (RNA-Seq) analysis. The six sequencing libraries produced an average of 6.28 G of data. A total of 26,419 genes were detected. The average alignment rate between the sequencing data and the rice genome was 92.38%, and the Q30 percentages were all greater than 85%. The results of transcription data are quantitatively verified by randomly selected genes (Supplementary Materials Figure S2). These results indicated that the RNA-Seq data were of sufficient quality to permit further analyses.

**Figure 4:**
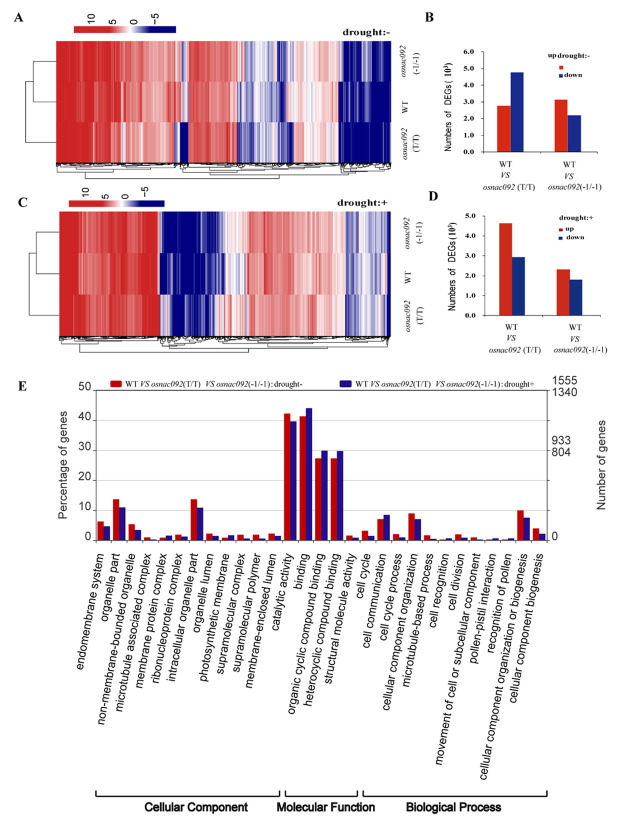
Knock out of *OsNAC092* results in genome-wide changes in plants. (**A**). WT and *osnac092* genome-wide expression analysis (drought−). (**C**). WT and *osnac092* genome-wide expression analysis (drought+). (**B**,**D**). Number of differentially expressed genes (DEGs) of WT and *osnac092*. (**E**). Gene ontology (GO) classification of DEGs shared by WT vs *osnac092* (T/T) vs *osnac092* (−1/−1):drought- and WT vs *osnac092* (T/T)vs *osnac092* (−1/−1):drought+.

Cluster analysis was used to show the transcriptional changes in knockout *OsNAC092* rice under normal conditions and drought treatment conditions. Under normal growth conditions, there were 7558 differentially expressed genes (DEGs) between *osnac092* (T/T) and the WT, of which 2782 were upregulated, and 4776 were downregulated. Between *osnac092* (−1/−1) and the WT, there were 5334 DEGs, including 3137 upregulated genes and 2197 downregulated genes (Figure 4A,B). Under drought stress conditions, there were 7583 DEGs between *osnac092* (T/T) and WT, including 4641 upregulated genes and 2942 downregulated genes. Between *osnac092* (−1/−1) and WT there were 4111 DEGs, including 2320 upregulated genes and 1791 downregulated genes (Figure 4C,D). The results showed that in *osnac092* (T/T) and *osnac092* (−1/−1), after drought treatment, more DEGs were upregulated and fewer were downregulated compared with those in the WT plants, which might improve the growth state of the whole mutant plant.

Venn diagram analysis showed the results of the analysis of the DEGs of the two mutants compared with the WT plants. There were 3111 DEGs under normal conditions and 2680 DEGs under drought stress (Supplementary Materials Figure S3). These DEGs were annotated into 29 gene ontology (GO) categories, including 14 cellular components (CC), 6 molecular functions (MF), and 19 biological processes (BP). The enrichment of DEGs in the BP category was significant, and the proportions of DEGs under normal conditions and drought conditions were 65.6% (2041/3111) and 65.7% (1760/2680), respectively. Signaling pathways, such as heterocyclic compound binding, organic cyclic compound binding, and catalytic activity, were altered. These data suggested that the knockout of *OsNAC092* affected the synthesis of organic compounds. In the CC category, biological processes, such as intracellular organelle function, photosynthetic membrane retention, and cell cycle process, were also affected, prompting us to speculate that the knockout of *OsNAC092* might affect the cell biomembrane system and the normal growth cycle (Figure 4E).

The Kyoto Encyclopedia of Genes and Genome (KEGG) annotation analysis showed the signaling pathways associated with the DEGs under normal conditions, as the “Biosynthesis of secondary metabolites.” The annotation results also found that “Plant hormone signal transduction,” and “MAPK signaling pathway,” were significantly altered (Supplementary Materials Figure S4). Under drought treatment, “photosynthesis,” “MAPK signaling pathways,” “plant hormone signal transduction,” “starch and sucrose metabolism,” “carotenoid biosynthesis,” and “drug, metabolism-cytochrome p450” pathways were enriched for the DEGs, and these biological processes play a role in drought regulation (Supplementary Materials Figure S5).

### 3.5. Analysis of DEGs Identified Possible Transcription Level Responses to Drought

Comprehensive GO annotation and Kyoto Encyclopedia of Genes and Genomes (KEGG) analysis revealed that the DEGs are more concentrated in “response to light stimulus,” “MAPK signaling pathway,” “plant hormone signal transduction,” “response to oxidative stress,” “photosynthesis,” and “water deprivation”.

**Figure 5:**
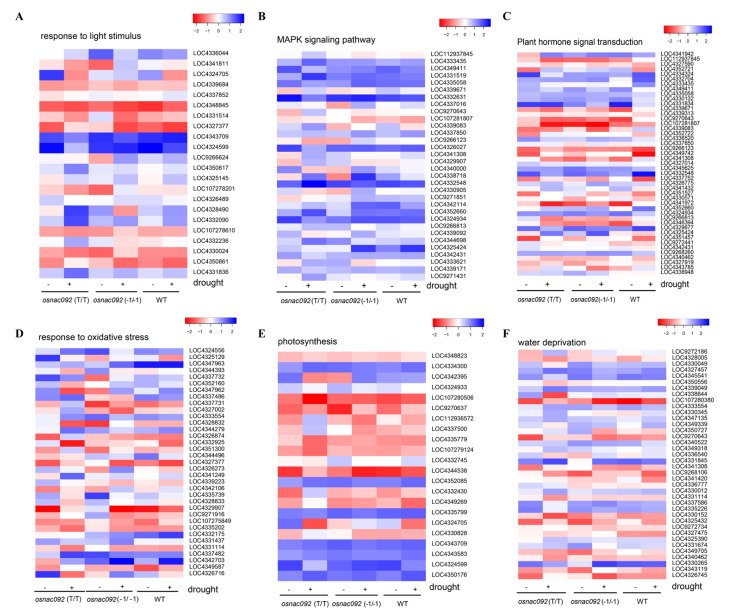
Transcriptome analysis of genes systemically regulated in the wild-type (WT) and *osnac092* T2 mutant lines in response to drought stress. (**A**). response to light stimulation. (**B**). MAPK signaling pathway. (**C**). response to oxidative stress. (**D**). photosynthesis. (**E**). plant hormone signal transduction. (**F**). water deprivation.

Heatmap analysis was then carried out for the most important genes in these six pathways. LOC4336044 encodes a protein GAPDH (glyceraldehyde-3-phosphate dehydrogenase) in the metabolic pathway of “light stimulation response” that is involved in carbon fixation and carbon metabolism in photosynthetic organisms. LOC4341811 encodes a protein G1L2 that is involved in the development of multicellular organisms (GO: 0007275) and postembryonic plant morphogenesis (GO: 0090698). LOC4331514 encodes a protein *OsJ_09298* which is involved in the recognition of endoplasmic reticulum targeting signals (GO: 0005786), chloroplast signal recognition (GO: 0009507), and chloroplast targeting signal recognition (GO: 0080085) pathways (Figure 5A) (Supplementary Materials Figure S6).

Many differentially expressed genes in the “MAPK signaling pathway” were also identified. For example, *SAPK10* (LOC4333435) and *SAPK4* (LOC4324934) encode proteins that are involved in biological processes such as protein kinase activity. The alteration of *SAPK3* (LOC4349411) expression directly affected protein phosphorylation, intercellular phosphorylation, and intracellular signal transduction. *OsETR2* (LOC4335058), encodes a protein that is involved in the regulation of ethylene-activated signaling pathways and starch metabolism. *OsPP2C51* (LOC4339671) encodes a protein that has been reported to regulate OsbZIP10 and is involved in regulating seed germination. *MPK2* (LOC4344698) encodes a protein that is involved in the targeted regulation of MAPK and is involved in the aging-related signaling pathways (Figure 5B) (Supplementary Materials Figure S7).

The expression levels of numerous genes encoding plant hormone pathway members that regulate auxin synthesis were altered. Genes such as *IAA30* (LOC4352721), IAA2 (LOC4326775), *IAA23* (LOC4341432), *IAA8* (LOC4330571), *IAA16* (LOC9272441), *IAA20* (LOC4327919), and *IAA12* (LOC4333512) encode proteins that belong to the AUX/IAA family, all of which have AUX/IA domains. *ARF14* (LOC4339313), *ARF3* (LOC4327014), and *ARF15* (LOC9268260) encode auxin response factors (ARFs) that participate in the auxin-activated signaling pathway (Figure 5C) (Supplementary Materials Figure S8).

For the response to oxidative stress pathway, alterations to *POXA* (LOC4347963) expression, which encodes an important component of the redox system, affects peroxidase activity (GO: 0004601), oxidation reductase activity (GO: 0016491), and other regulatory enzyme activities. The gene encoding peroxidase 72 (LOC4325129) was also a DEG which participates in the decomposition of hydrogen peroxide (GO: 0042744) (Figure 5D). For this pathway, all differential genes are basically located around the peroxisome synthesis pathway; therefore, the peroxisome is affected after knockout *OsNAC092* (Supplementary Materials Table S4).

The seven light-harvesting genes (LOC4324705, LOC4330828, LOC4343709, LOC4343583, LOC4324599, LOC4350176, and LOC4335799), belonging to the chlorophyll a/b superfamily, were upregulated, thus the knockout of *OsNAC092* might directly promote light harvesting in photosystem I (Figure 5E) (Supplementary Materials Figure S9).

Analysis of the signaling pathways of water-deficient stress found that *PIP2-2* (LOC4330049) and *PIP2-6* (LOC4335226), which encode plasma membrane intrinsic proteins, are important components of aquaporins and are involved in the vacuoles (GO: 0005773) and plasma membranes (GO: 0005886) (Figure 5F).

### 3.6. Knockout of OsNAC092 Resulted in Changes in Antioxidant Content and Enzymatic Activities Related to the ROS Pathway

**Figure 6:**
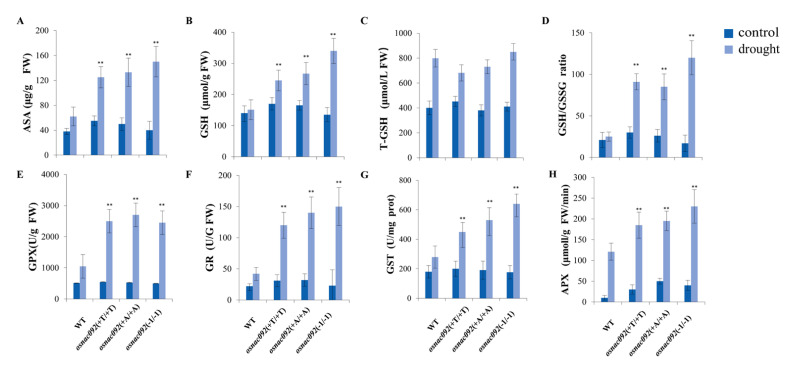
Antioxidant contents and activity of enzymes in the ascorbate-glutathione cycle were shown in wide type (WT) and *osnac092* mutant plants. (**A**) ASA. (**B**) GSH content. (**C**) T-GSH content. (**D**) the ratio of GSH/GSSG. (**E**) GPX. (**F**) GR. (**G**) GST. (**H**) APX. ** represent significant differences at *p* < 0.01 compared with wide type. The detection of antioxidant contents and activity of enzymes 8-week-old WT and mutants under 14 days drought treatment.

Through the analysis of transcriptome data, we clearly found that after knocking out *OsNAC092*, the redox system changed greatly. The KEGG pathway display DEGs are involved in ROS metabolism, peroxisomal division, the receptor recycling of peroxisome biogenesis, and oxidative phosphorylation (Supplementary Materials Figure S10). The significantly differentially expressed genes are almost all concentrated in the genes related to peroxisome synthesis (Supplementary Materials Table S1). In order to further study the effect of drought on the growth of mutants, several important physiological indicators of both the wild-type and mutant rice plants were determined, including the content of key components in the glutathione-ascorbic acid cycle and related enzyme activities. Under drought stress, the contents of ascorbic acid, glutathione, total glutathione, and the activities of the antioxidant enzymes (GPX, APX, and GR) were all higher than those of the wild type (Figure 6A–G). In addition, the GSH/GSSG ratio of the mutant line was also significantly higher than that of the wild type (Figure 6D). These results indicate that *osnac092* can scavenge more ROS under stress conditions, maintain a relatively high intracellular redox level, and provide favorable conditions for its growth.

## 4. Discussion

Drought causes cellular disorders by depriving the cells of water and nutrients, reducing crop productivity more than any other environmental stress. Plant tolerance to dehydration is complex and varies widely at the molecular and physiological levels at different developmental stages, thus presenting many research challenges [23]. Therefore, as transcription factors related to adverse stress, the NAC transcription factor family has become a hot topic in research [24]. Rice *OsNAC2* transcription factor CRISPR/Cas9 plants showed better drought and salt resistance, indicating that OsNAC2 is a transcription factor that negatively regulates drought and high salt stress responses [25,26,27,28,29]. Wu et al. found that *PtrNAC72* in the Chinese wolfberry (*Poncirus trifoliata*) is a repressor of putrescine biosynthesis, and it may negatively regulate the drought stress response by regulating putrescine-related active oxygen homeostasis [30]. Due to the limitation of technical resources, a small number of NAC transcription factors reported in the family have negative regulatory effects in the process of stress regulation. Our focus on *OsNAC092* has filled some gaps.

In the current study, organ expression analysis found that the expression of *OsNAC092* was most significantly induced by PEG-simulated drought stress (Figure 1). Analysis of its promoter sequence identified a large number of drought-responsive elements, suggesting that OsNAC092 is related to drought regulation. Therefore, we chose to design an sgRNA for the 5′ end of the second exon to obtain mutants for further study. From the test results, the mutation efficiency was as high as 90%, and most of them are single-base insertion mutations (Figure 2). We conducted stress observations on the mutants and found that they have better drought resistance at both the seedling and the heading stages.

Photosynthesis is the most fundamental and intricate physiological process occurring in plants. Photosynthetic mechanisms include photosynthetic pigments, different types of which are present in varying abundances in photosynthetic systems [31,32,33]. In the present study, *osanc092* plants showed a higher chlorophyll content after drought treatment (Figure 3), which might be partly responsible for the observed higher Pn under drought conditions in the mutant lines. In the mutant lines, higher levels of ROS peroxisome protection enzymes were detected under stress. ROS, which are highly toxic and cause damage to proteins, lipids, and DNA, include superoxide radicals (O^2−^), hydrogen peroxide (H_2_O_2_), single oxygen (O_2_^1^), alkoxy radicals (RO), and hydroxyl radicals (OH^−^) [34]. MDA levels are a well-known indicator to determine the degree of lipid peroxidation caused by ROS [35]. A higher MDA content was detected in the WT plants after drought stress, indicating that these plants were suffering from more oxidative damage. In response to ROS toxicity, plants have evolved efficient antioxidant mechanisms [36]. These mechanisms mainly include non-enzymatic scavengers and antioxidative enzymes. Non-enzymatic antioxidants include ascorbic acid and reduced glutathione, and enzymatic antioxidants include superoxide dismutase (SOD), catalase (CAT), and peroxidase (POD), which under drought conditions, can accumulate rapidly to minimize oxidative damage [37]. The *osnac092* plants showed higher SOD, CAT, and POD activities than the WT plants following drought stress, suggesting that the mutants obtain higher drought resistance by regulating ROS pathway-related genes (Figure 3).

To reveal how *OsNAC92* works with other genes to enhance drought tolerance, we used expression profiling to identify genes that were differentially expressed in the WT and mutant plants (Figure 4). The results suggested that *OsNAC092* might activate the expression of genes involved in photosynthesis, the MAPK signaling pathway, and protein protection (Figure 5). We found that many photosynthesis-related genes were upregulated in the *osnac092* plants in response to drought, suggesting that *OsNAC092* enhances the plant’s photosynthetic capacity by regulating the expression of related genes [38]. It has been reported that phytohormones play a pivotal role in almost every aspect of plant development [39,40]. Hormones are simple, small-molecule organic compounds that have important regulatory and control effects on plant stress; however, their physiological effects are very complex and diverse [41,42]. Low auxin concentrations can regulate gene expression via certain TFs and regulate proteins that respond to environmental changes via signaling cascades [43]. Tissues with high cell division activity synthesize auxin, whose efflux and influx is controlled by specific transmembrane proteins [44,45,46]. Our results also indicated that hormone-related gene expression of the IAA and ARF signaling pathways were significantly changed in our mutants (Figure 5). Aquaporins PIP2-2 and PIP2-6 are important in the drought stress response, in which the ERAD component ubiquitin-conjugating enzyme UBC32 regulates drought tolerance positively in *Arabidopsis thaliana* by targeting PIP2-2 for degradation [47]. PIP2-2 and PIP2-6 were significantly differentially expressed in the *osnac092* plants (Figure 5).

Moreover, the redox pathway in plants has been reported to reduce plant damage. Total glutathione (T-GSH) in plants includes reduced glutathione and oxidized glutathione. Changes in the external environment have a very significant impact on the content of T-GSH. The external application of other stress conditions such as heat, drought, and salt, can increase the T-GSH pool of plants, and conversely, plants with high T-GSH contents also show tolerance to environmental stress [48].

The conversion between reduced glutathione (GSH) and oxidized glutathione (GSSG) is carried out by three enzymes: glutathione peroxidase (GPX), glutathione reductase (GR), and glutathione S-transferase (GST). In addition to participating in the glutathione-ascorbic acid cycle, glutathione can also reduce other types of peroxides, such as membranous peroxides, glyoxal, and herbicides, through GPX [18]. In addition, GSH can not only directly scavenge ROS accumulated in plants under adverse environments, but it also activates multiple downstream defense systems to indirectly protect cells from damage [15,49,50]. For example, GSH can interact with the redox molecules thioredoxin and glutaredoxin, and the plant hormones acetylsalicylic acid and abscisic acid, to regulate gene expression and protein activity [51]. Glutathione, as the main and largest sulfhydryl pool in plants, is the main method of reducing sulfur storage and transport [52,53]. In our data, the expression of GSH, GR, and GST were all significantly increased under drought stress; therefore, we have sufficient experimental data to prove that the mutants have a higher ROS scavenging ability.

At the same time, glutathione mainly depends on the ratio of GSH/GSSG to regulate the redox state in tissues, which shows specificity for time and space. Glutathione accumulates by increasing the activity of glutathione reductase and inhibiting the activity of glutathione peroxidase. The mutants in our assay had significantly higher GSH/GSSG ratios, which is beneficial to plants. Glutathione reductase (GR) is a key enzyme in the glutathione-ascorbic acid cycle. GR also showed higher expression levels in our experiments, and we speculated that the mutants have better antioxidant properties.

The ROS system affects the photosynthetic mitogen-activated protein kinase (MAPK) signaling pathway, plant hormones, and dehydration responses. ROS also affect the assembly, repair, and chloroplast development of PSII, which will affect the early development of leaves and chloroplasts [54]. The mediators linking chloroplast ROS signaling and cytoplasmic signaling are MAPK and serine/threonine protein kinase OXI1. Among these, MAPK is related to the stress response and adaptation of plants to stress, which in turn can regulate the homeostasis of intracellular ROS [55,56,57]. For example, the MAPKKK-like gene *MEKK1* is involved in plant hormone signal transduction and the regulation of intracellular redox status [57]. The overexpression of MEK2 in tobacco can cause programmed cell death similar to the hypersensitivity reaction in transgenic plants, resulting in the destruction of chloroplast structure, the depolarization of the plasma membrane, and the loss of the intracellular component [58]. ROS and GSH/GSSG are closely related to plant hormone signal transduction pathways, such as acetylsalicylic acid (SA), abscisic acid (ABA), and jasmonic acid (JA) [59]. The exogenous application of SA to maize reduced catalase activity, promoted H_2_O_2_ accumulation, and increased tolerance to cold stress. The application of exogenous SA to Arabidopsis also promoted the formation of H_2_O_2_ and improved the tolerance to salt and oxidative stress [60]. In addition, SA can also increase the content of GSH and γ-EC, the enzymatic activity and expression level of GR and γ-ECS, and accelerate the synthesis and accumulation of glutathione [61].

Therefore, our results show that changes in “response to light stimulus,” “MAPK signaling pathway,” “plant hormone signal transduction,” “response to oxidative stress,” “photosynthesis,” and “water deprivation” pathways are likely to represent the combined action that eventually leads to the transformation of the ROS redox system, which in turn leads to *OsNAC092* directly or indirectly protecting plants from drought stress by enhancing the activity of antioxidants (Figure 6).

## 5. Conclusions

**Figure 7:**
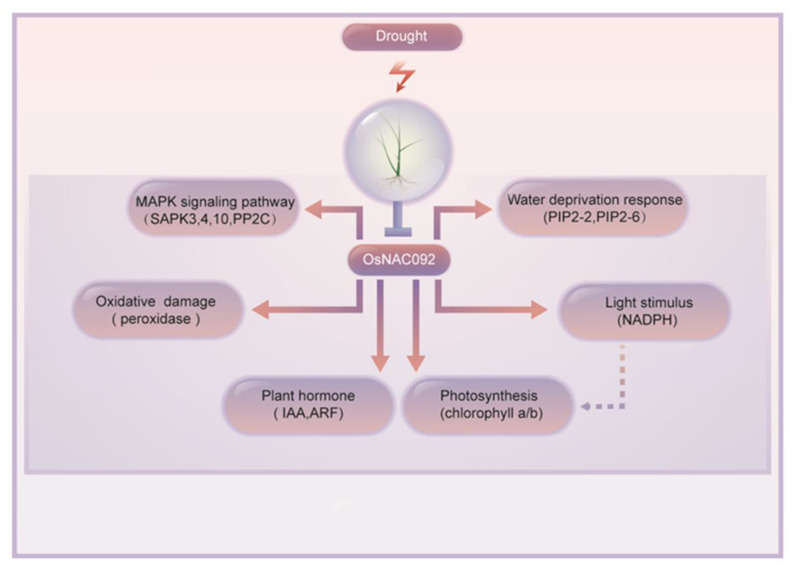
Predicted regulatory network of *OsNAC092* under drought stress.

The findings of the present study suggested that *OsNAC092* (Os06g0675600) has vital functions for rice drought stress. Knockout *OsNAC092* increased rice drought tolerance. Transcriptomic analysis showed that the DEGs between the WT and mutant lines under drought stress mainly affected the processes of ROS response, photosynthesis, light stimulus, and the plant hormone and MAPK signal pathway (Figure 7). The antioxidant contents and activity of enzymes in the ascorbate-glutathione cycle were highly expressed in the *osnac092* mutant plants to promote rice drought tolerance. The above experimental results clearly indicated the metabolic pathways involving *OsNAC092*, and further analysis of their regulation under drought stress will promote the molecular breeding of drought tolerant rice.

## Data Availability

Transcriptome raw data has been uploaded to the National Center for Biotechnology Information (NCBI) BioProject database; the accession number is PRJNA864565.

## Supplementary Materials

The following supporting information can be downloaded at: https://www.mdpi.com/article/10.3390/biology11121830/s1, Supplementary Materials Table S1: Promoter sequence analysis; Supplementary Materials Table S2: Off-target analysis of *OsNAC092*-sgRNA01; Supplementary Materials Table S3: Gene description of response to oxidative response; Supplementary Materials Table S4: Oligonucleotides used in this study; Supplementary Materials Figure S1: Amino acid sequence analysis of osnac092 mutants; Supplementary Materials Figure S2: Validation of the RNA-seq results by qRT-PCR; Supplementary Materials Figure S3: Venn diagram of the DEGs; Supplementary Materials Figure S4: KEGG analysis of WT vs. *osnac092* (T/T) vs. *osnac092* (−1/−1): drought-DEGs; Supplementary Materials Figure S5: KEGG analysis of WT vs. *osnac092* (T/T) vs. *osnac092* (−1/−1): drought+ DEGs; Supplementary Materials Figure S6: Schematic diagram of DEGs related to photosynthesis-antenna proteins; Supplementary Materials Figure S7: Schematic diagram of DEGs related to MAPK signal transduction pathway; Supplementary Materials Figure S8: schematic diagram of DEGs related to plant hormone signal transduction; Supplementary Materials Figure S9: Schematic diagram of DEGs related to photosynthesis; Supplementary Materials Figure S10: Schematic diagram of DEGs related to peroxisomes.
