# Supplementary material for: Knocking Out the Transcription Factor OsNAC092 Promoted Rice Drought Tolerance
Source: Biology (Basel). 2022 Dec 15;11(12):1830. doi: 10.3390/biology11121830 (PMC9776343; doi:10.3390/biology11121830)
Supplement: Supplementary file 1 [file biology-11-01830-s001.zip › biology-2049015-supplementary figures.pptx]

## Slide 1
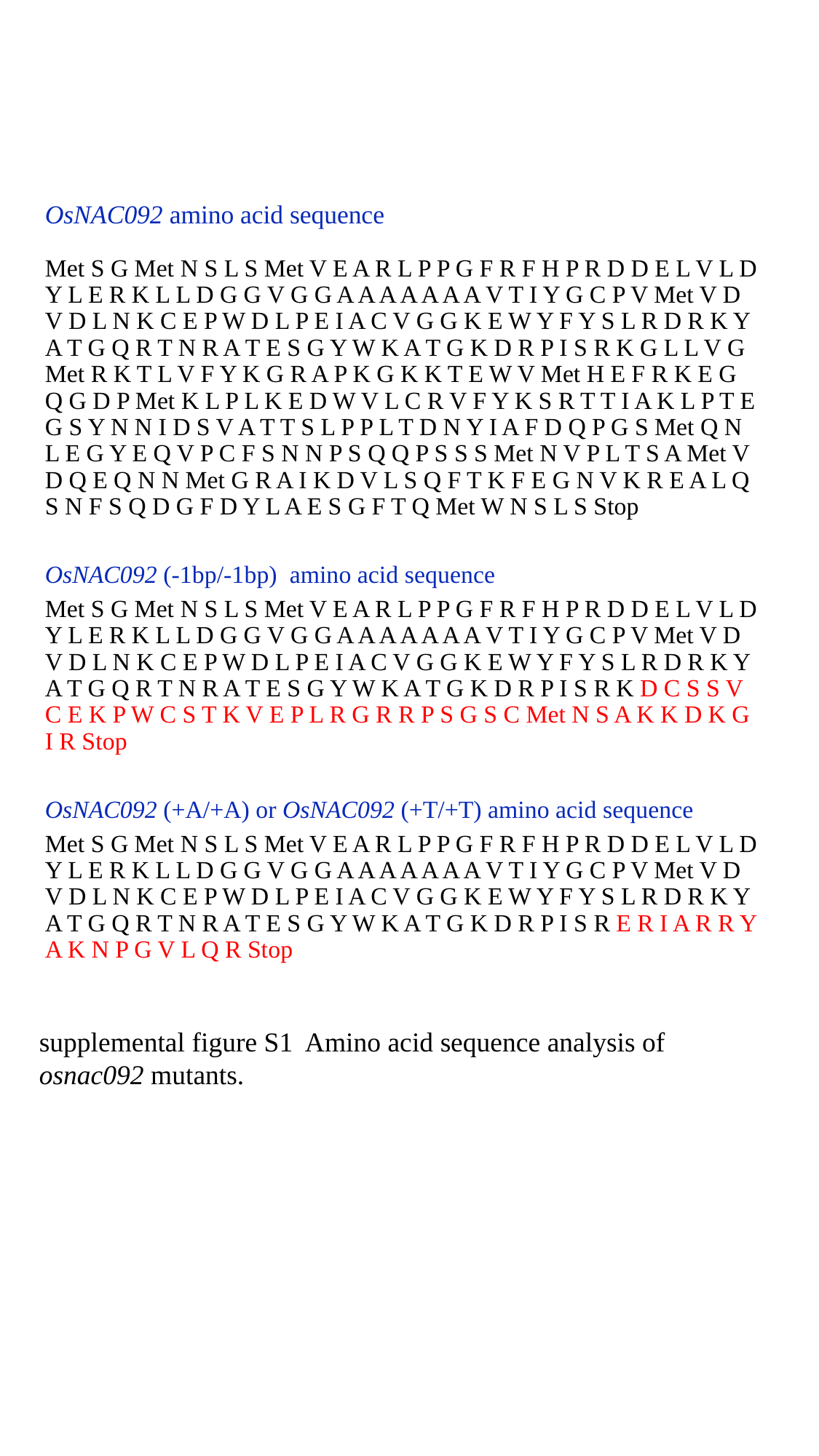

OsNAC092 amino acid sequence
Met S G Met N S L S Met V E A R L P P G F R F H P R D D E L V L D Y L E R K L L D G G V G G A A A A A A A V T I Y G C P V Met V D V D L N K C E P W D L P E I A C V G G K E W Y F Y S L R D R K Y A T G Q R T N R A T E S G Y W K A T G K D R P I S R K G L L V G Met R K T L V F Y K G R A P K G K K T E W V Met H E F R K E G Q G D P Met K L P L K E D W V L C R V F Y K S R T T I A K L P T E G S Y N N I D S V A T T S L P P L T D N Y I A F D Q P G S Met Q N L E G Y E Q V P C F S N N P S Q Q P S S S Met N V P L T S A Met V D Q E Q N N Met G R A I K D V L S Q F T K F E G N V K R E A L Q S N F S Q D G F D Y L A E S G F T Q Met W N S L S Stop
OsNAC092 (-1bp/-1bp) amino acid sequence
Met S G Met N S L S Met V E A R L P P G F R F H P R D D E L V L D Y L E R K L L D G G V G G A A A A A A A V T I Y G C P V Met V D V D L N K C E P W D L P E I A C V G G K E W Y F Y S L R D R K Y A T G Q R T N R A T E S G Y W K A T G K D R P I S R K D C S S V C E K P W C S T K V E P L R G R R P S G S C Met N S A K K D K G I R Stop
OsNAC092 (+A/+A) or OsNAC092 (+T/+T) amino acid sequence
Met S G Met N S L S Met V E A R L P P G F R F H P R D D E L V L D Y L E R K L L D G G V G G A A A A A A A V T I Y G C P V Met V D V D L N K C E P W D L P E I A C V G G K E W Y F Y S L R D R K Y A T G Q R T N R A T E S G Y W K A T G K D R P I S R E R I A R R Y A K N P G V L Q R Stop
supplemental figure S1 Amino acid sequence analysis of osnac092 mutants.

## Slide 2
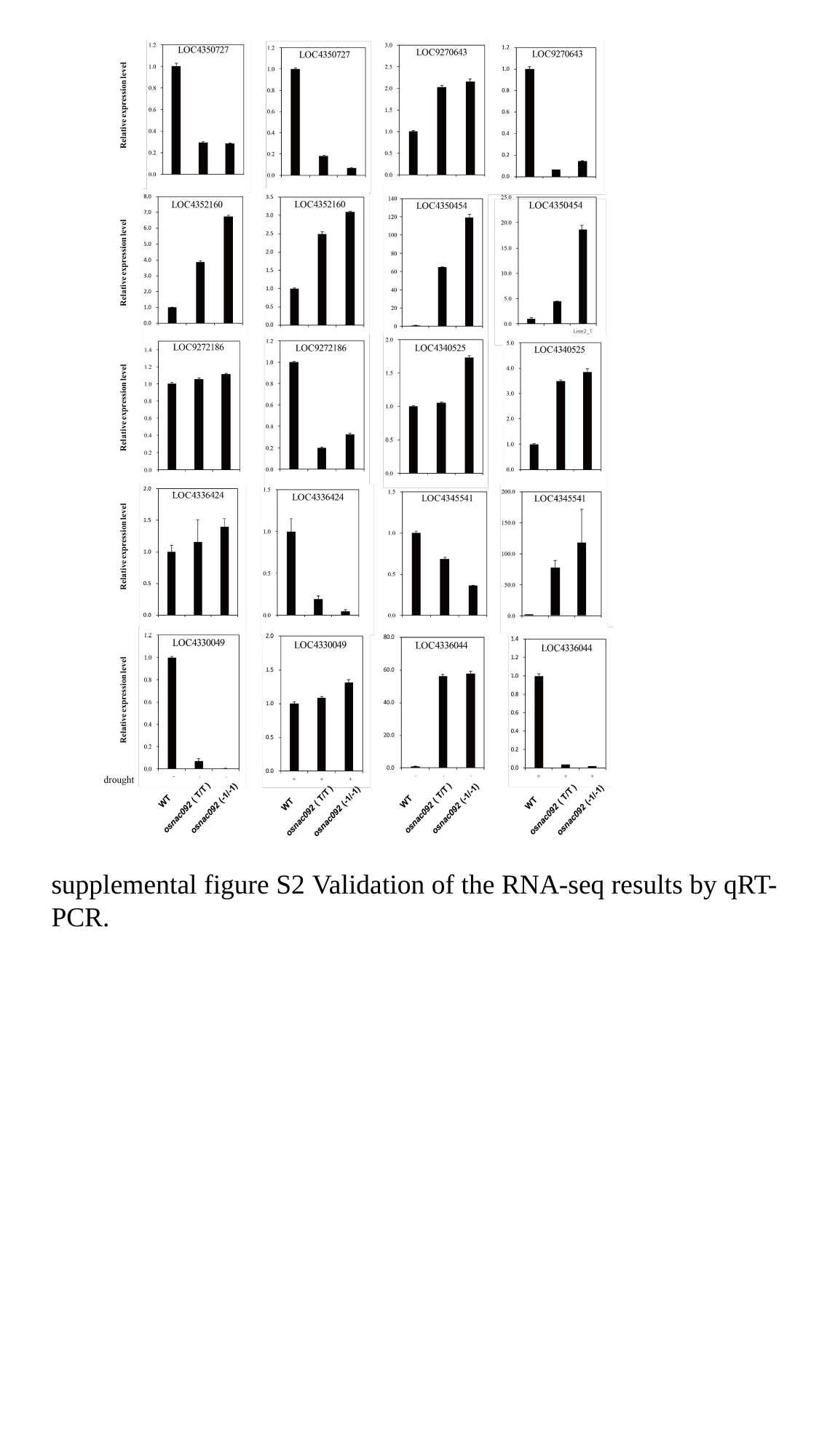

supplemental figure S2 Validation of the RNA-seq results by qRT-PCR.

## Slide 3
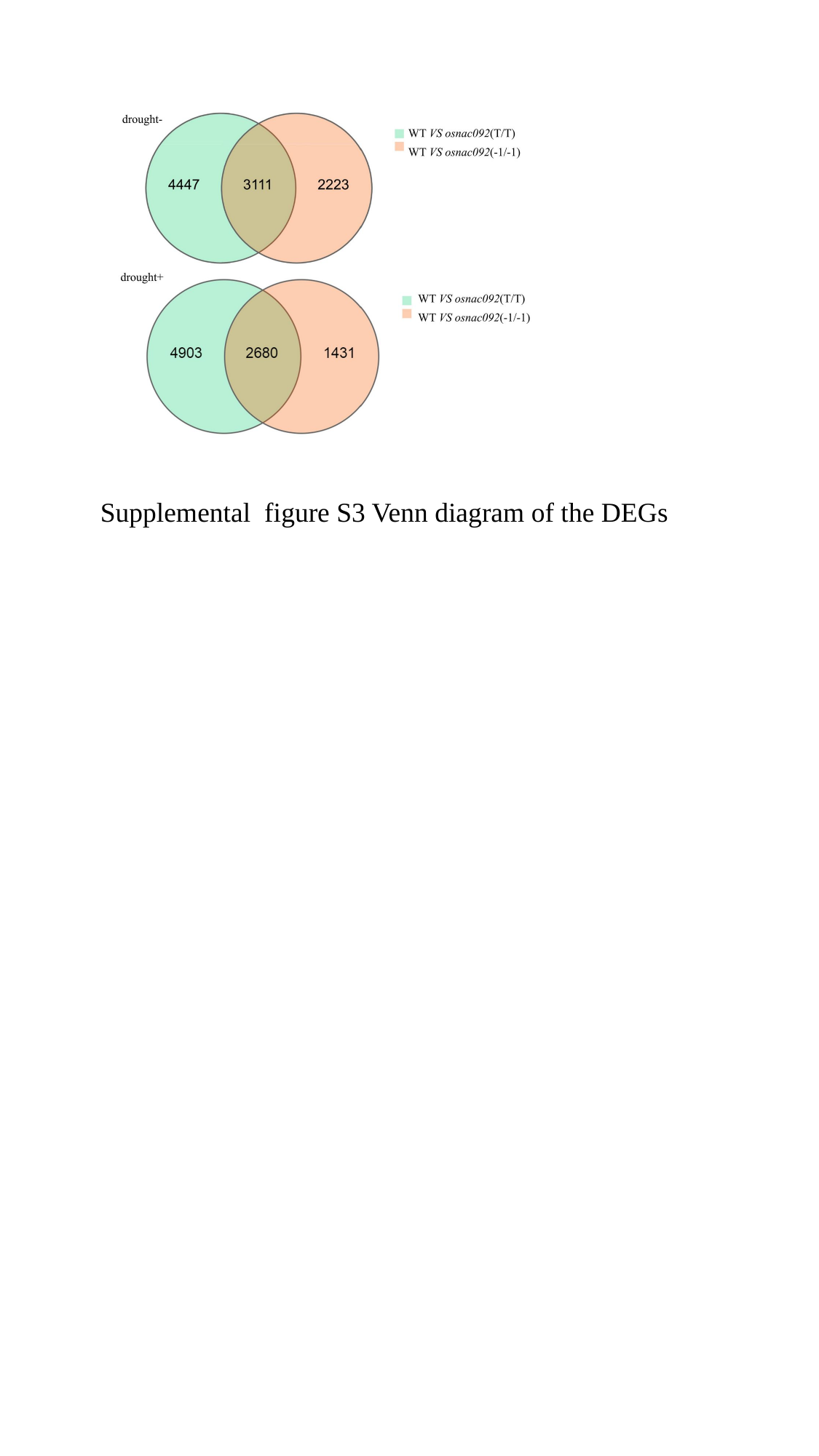

Supplemental figure S3 Venn diagram of the DEGs

## Slide 4
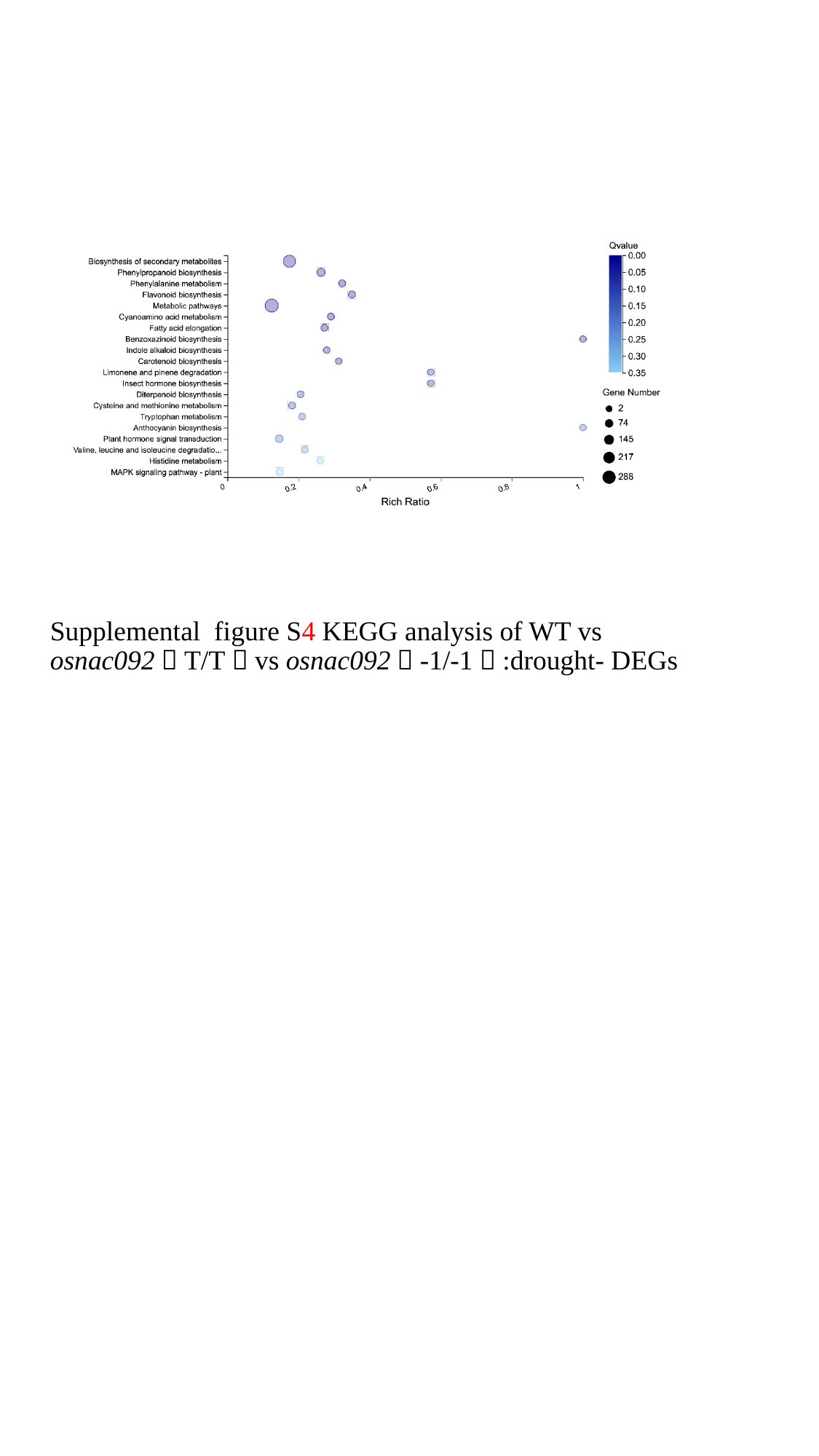

# Supplemental figure S4 KEGG analysis of WT vs osnac092（T/T）vs osnac092（-1/-1）:drought- DEGs

## Slide 5
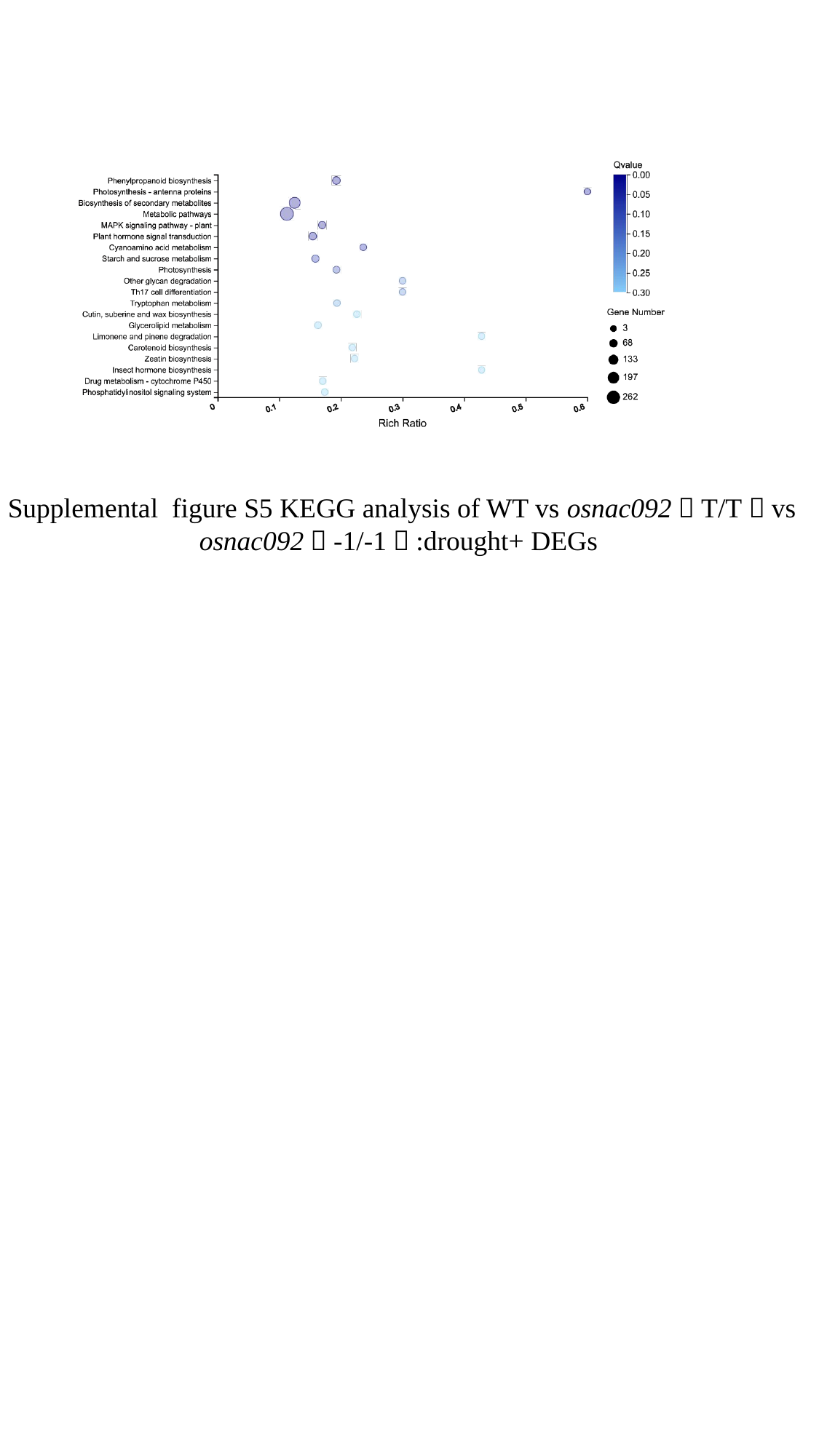

Supplemental figure S5 KEGG analysis of WT vs osnac092（T/T）vs osnac092（-1/-1）:drought+ DEGs

## Slide 6
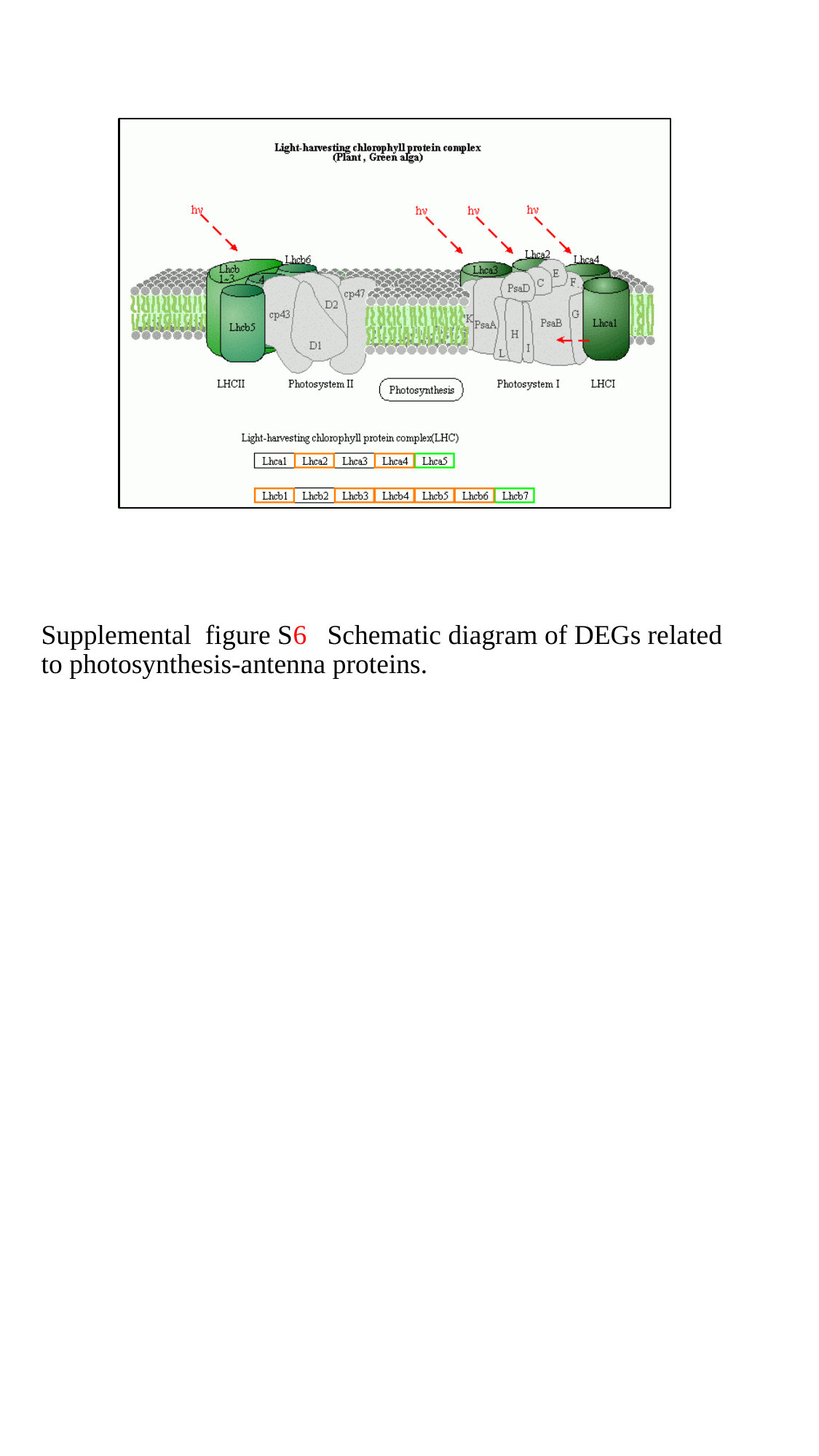

# Supplemental figure S6 Schematic diagram of DEGs related to photosynthesis-antenna proteins.

## Slide 7
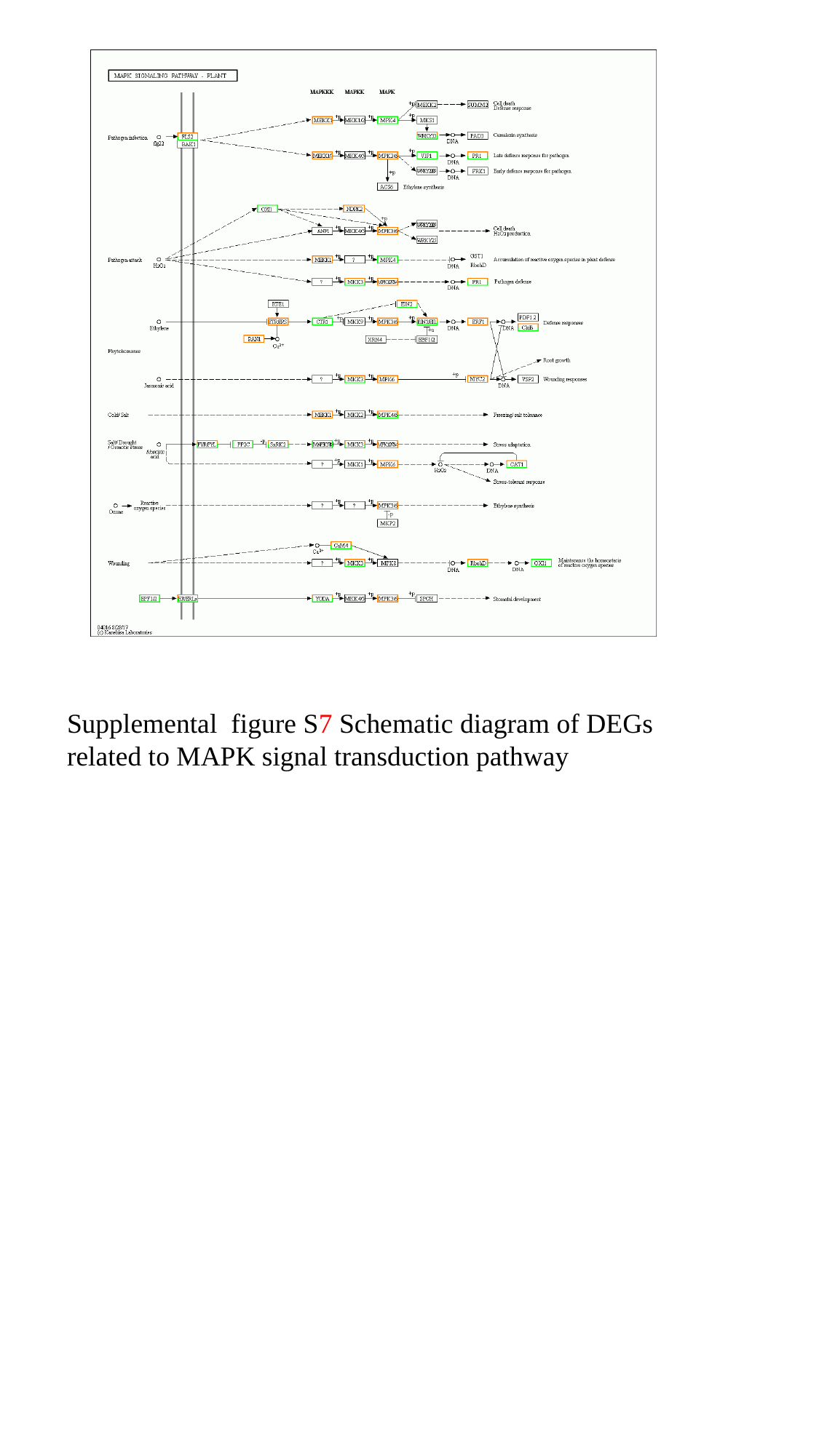

#
Supplemental figure S7 Schematic diagram of DEGs related to MAPK signal transduction pathway

## Slide 8
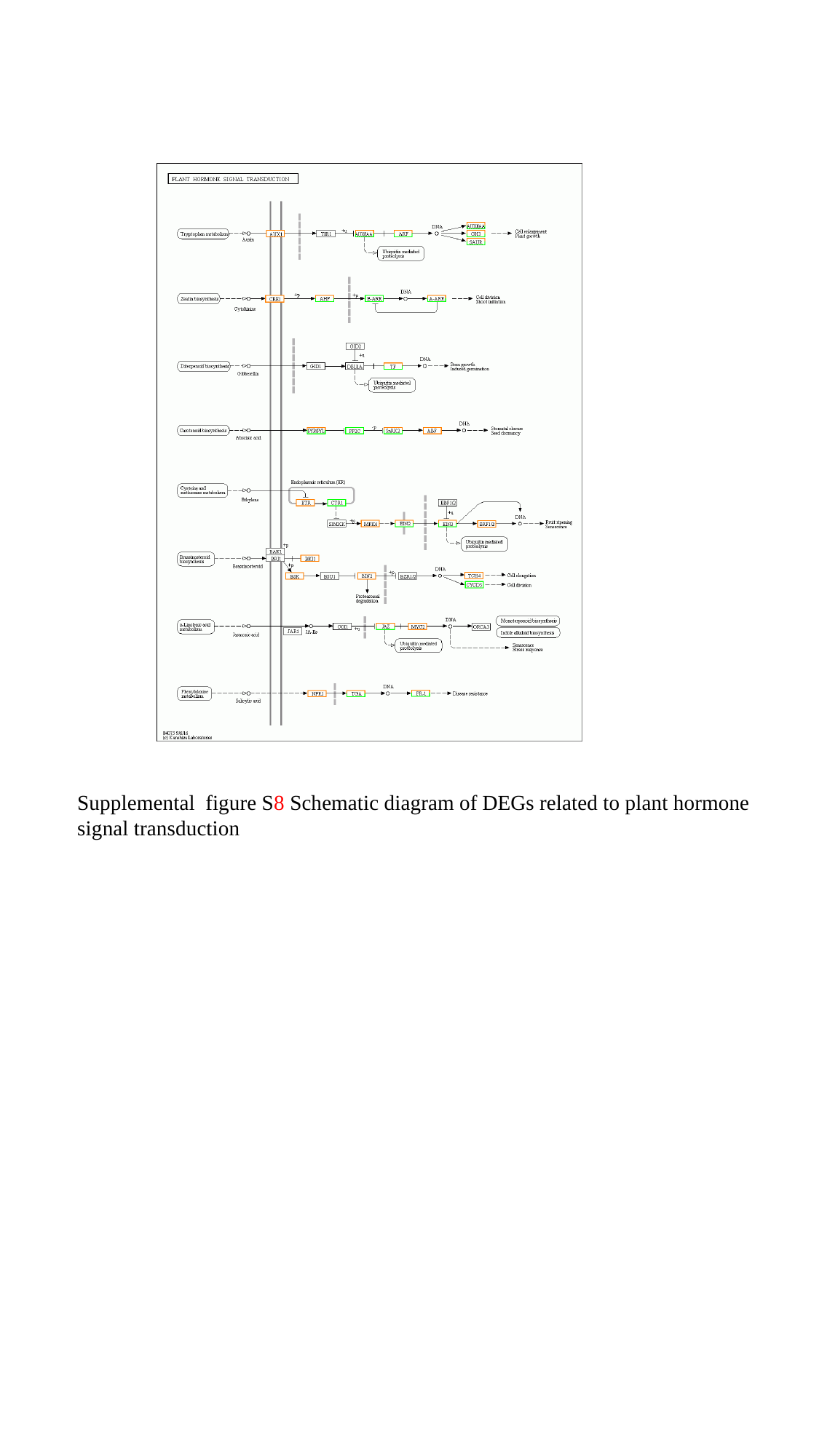

#
Supplemental figure S8 Schematic diagram of DEGs related to plant hormone signal transduction

## Slide 9
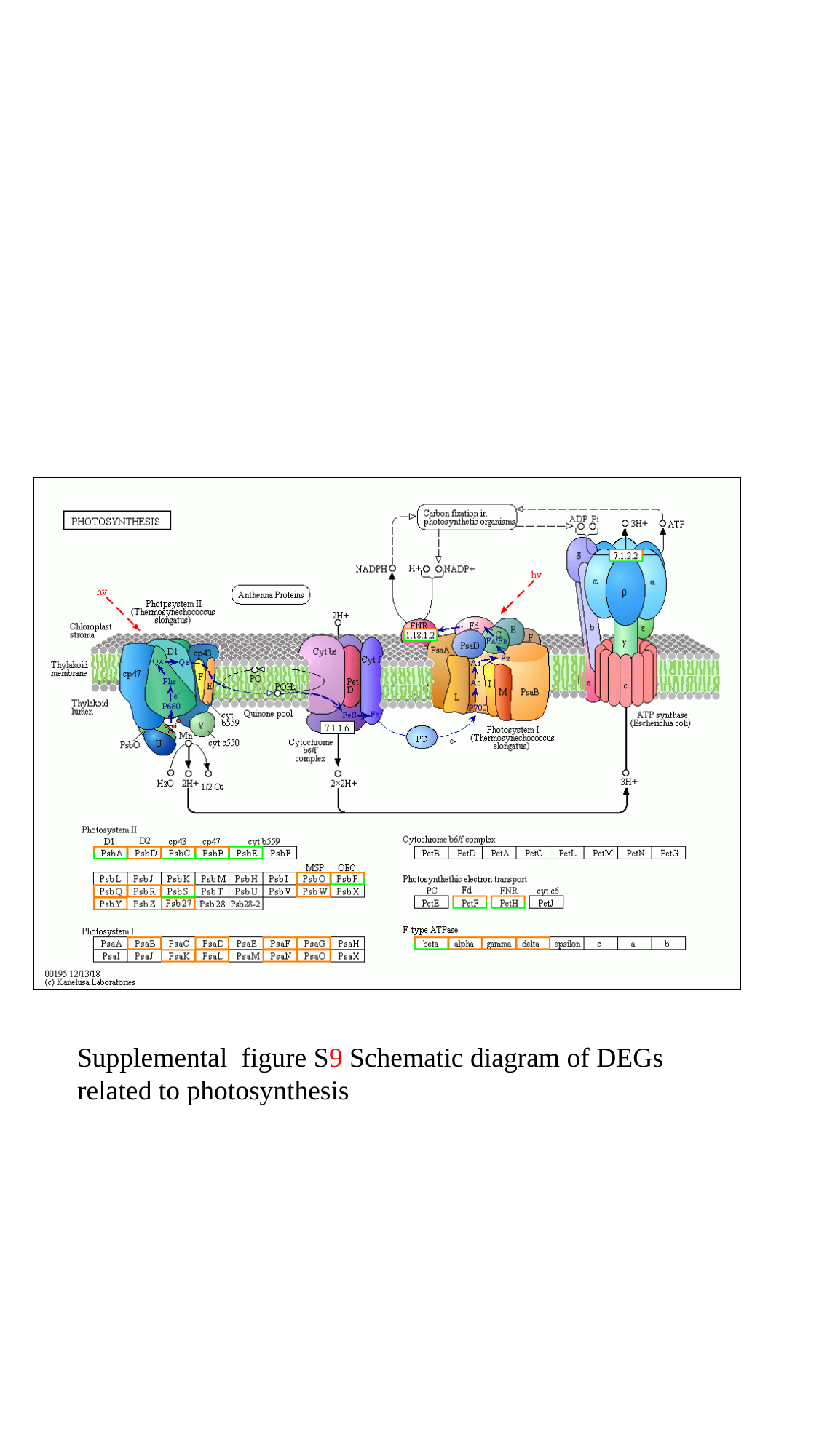

#
Supplemental figure S9 Schematic diagram of DEGs related to photosynthesis

## Slide 10
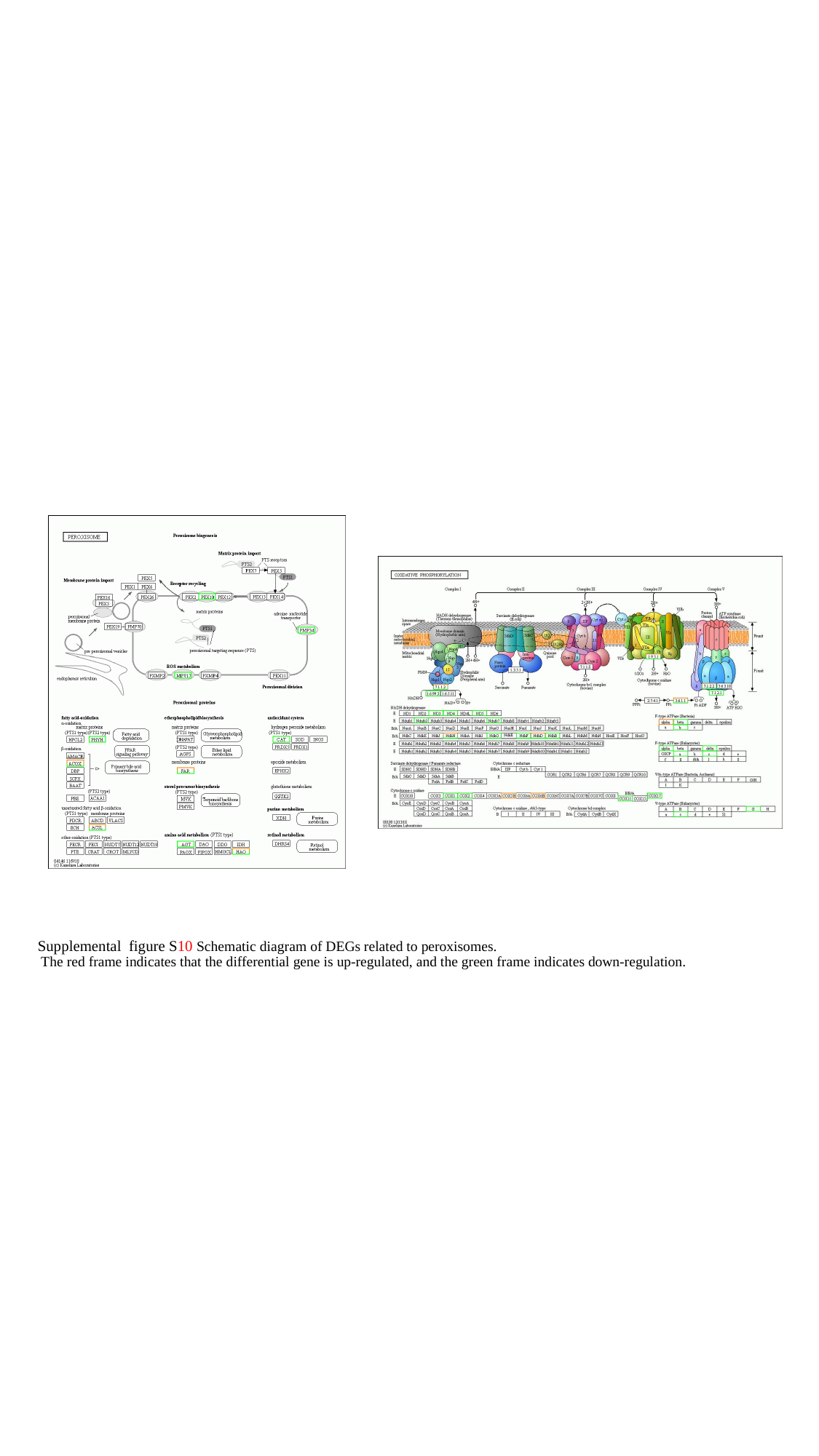

# Supplemental figure S10 Schematic diagram of DEGs related to peroxisomes. The red frame indicates that the differential gene is up-regulated, and the green frame indicates down-regulation.
