# Supplementary material for: Knocking Out the Transcription Factor OsNAC092 Promoted Rice Drought Tolerance
Source: Biology (Basel). 2022 Dec 15;11(12):1830. doi: 10.3390/biology11121830 (PMC9776343; doi:10.3390/biology11121830)
Supplement: Supplementary file 1 [file biology-11-01830-s001.zip › biology-2049015-supplementary tables.pdf]

Supplemental table S1 Promoter sequence analysis

Factor or Site Name	Signal Sequence	Reference	Number of binding sites
WRKY71	TGAC	Involved in gibberellin signaling pathway in rice, affecting abscission	12
MYBCORE	CNGTTR	Involved in dehydration in Arabidopsis	8
MYB2consensus	YAACKG	Direct regulation of RD22 dehydration-responsive genes in Arabidopsis	3
MYB2	TAACTG	Involved in dehydration signaling pathway in Arabidopsis	1
ERD1	ACGT	ACGT sequence induces erd1 gene expression in Arabidopsis	8
DOF	AAAG	Dof protein in maize affects growth and development	26
GAPB	ACTTTG	GAPB encodes the chloroplast glyceraldehyde-3-phosphate dehydrogenation (GADPH) B subunit	4
MYCCONSENSUSAT	CANNTG	MYC can interact with *rd22*, *CBF3* and *ICE1*	18

Supplemental table S2 off-target analysis of *OsNAC092*-sgRNA01

Target	Chromosome	Position	Direction	Mismatches	Examined lines number	Off-targets number
**GATCGgCCgAaAcGCCGGAACGG**	chr3	3788628	-	4	8	0
**GATCGCCCAtgAgGCCGcAACGG**	chr3	33750481	+	4	8	0
**cATCGCgCAATAAcaCGGAATGG**	chr4	14331158	+	4	8	0
**cATCGCgCAATAAcaCGGAATGG**	chr4	14892707	-	4	8	0
**cATgGCgCAATAAGCCGGcAGGG**	chr4	22882929	-	4	8	0
**GATCGaCCAATAAGgCGatATGG**	chr2	7684332	-	4	8	0
**GActGCCCgAgAAGCCGGAAAGG**	chr10	18972232	-	4	8	0
**GATCtCCaAATgAGCCGaAATGG**	chr9	17543867	+	4	8	0
**GtTgGCCCAgcAAGCCGGcATGG**	chr8	5438116	-	4	8	0
**GATttCCCAATAAGgaGtAATGG**	chr8	12977895	+	4	8	0

Supplemental table S3 Gene description of response to oxidative response

Gene Symbol	Gene description
LOC4324556	cationic peroxidase SPC4
LOC4325129	peroxidase 72
LOC4347963	peroxidase N
LOC4344393	peroxidase 21
LOC4337732	peroxidase 5
LOC4352160	alpha-dioxygenase 1
LOC4347962	peroxidase A2
LOC4337486	cationic peroxidase SPC4
LOC4342185	peroxidase 1-like
LOC4337731	peroxidase 5
LOC4337483	cationic peroxidase SPC4
LOC4327002	peroxidase 1
LOC4333554	ornithine aminotransferase, mitochondrial-like
LOC4328832	peroxidase P7
LOC4344279	Peroxidase 2
LOC4326874	peroxidase 72
LOC4332925	peroxidase 2
LOC4351300	peroxidase 4
LOC4344496	peroxidase 47
LOC4327377	zinc finger protein ZAT12
LOC4326273	peroxidase 24
LOC4341249	peroxidase P7
LOC107280449	peroxidase 5-like
LOC4339223	peroxidase 1-like
LOC4342106	thiosulfate sulfurtransferase 16, chloroplastic
LOC4335739	uncharacterized protein At1g32220, chloroplastic
LOC4337232	peroxidase 4
LOC4328841	peroxidase 70
LOC4328833	peroxidase P7
LOC4329907	serine/threonine-protein kinase OXI1
LOC9271916	probable galactinol--sucrose galactosyltransferase 2
LOC107275849	cationic peroxidase 1
LOC4335202	probable L-ascorbate peroxidase 3, peroxisomal
LOC4332175	peroxidase A2
LOC4331437	peroxidase 5
LOC4331114	transcriptional corepressor LEUNIG_HOMOLOG
LOC4337482	cationic peroxidase SPC4
LOC4342703	probable galactinol--sucrose galactosyltransferase 2
LOC4349587	peroxidase 4
LOC4326716	peroxidase 2

Supplemental table S4 Oligonucleotides used in this study

primer name	forward primer（5′→ 3′）	reverse primer（5′→ 3′）
OsActin	AGCTGCGGGTATCCATGAGA	GCAATGCCAGGGAACATAGTG
DCAS9	TCTTCTCACCAGGGAGCTGAGCA	CATATGCAGCAGCTATATGTGGA
pZHY988	GGGCTGATCCTAAGAAGAAGAGGAA	TTCTAATAAACGCTCTTTTCTCT
OsNAC050	GTCGCAGTCGCACTCGCACACCCAC	CCAGCTCCGGGAAGGGGTCGTTGTC
4325129	CCTCTTCCCGCTAGACTTT	CTTGCCAGACAGGATGTTC
4332851	TGTTGATGATGGCGAGAA	ATCAGCCACATTGGAAGG
4340349	TTGCCTGGCTATGATACTCT	TGTGCTTAGTGCCTTCCTC
4325401	ACACCGACATGGAGGTTGA	AGAGGCGAAGAGGAAGCA
4343814	CGGCAGGGTCAAGAGTTCG	CGTCTCGCTGGAGGAGTTCG
4346335	GCTACCTCTTCACTCGCTCACA	CGCCATTGTCGTCGTCGTT
4347135	CGCATCTCGGGGCTCATC	GCGTGCTCGGTGTAGGTGAC
4340632	TGACAAGGCTGTGAGACA	ATCGGAGACGAAGAGGAG
4325129	CCTCTTCCCGCTAGACTTT	CTTGCCAGACAGGATGTTC
4332851	TGTTGATGATGGCGAGAA	ATCAGCCACATTGGAAGG
